# Artisanal Sour Starch from Peruvian *Manihot esculenta* Ecotypes: Physical, Chemical, and Functional Properties

**DOI:** 10.3390/foods15213780

**Published:** 2026-10-22

**Authors:** Thony Arce-Saavedra, Elis Delgado-Marrufo, Lusmenia Ruiz-Mejía, Flor de María Valqui-Perez, Yajaira Gavidia-Perez, Gabriela Barreto-Tarrillo, Blanca L. Cabrera-Campos, José L. Marcelo-Peña, Katherin L. Arce-Rios, Tony Chuquizuta

**Affiliations:** 1 Grupo de Investigación en Productos y Procesos Agroindustriales, Facultad de Ciencias Agrarias, Universidad Nacional Autónoma de Chota (UNACH), Chota 06121, Peru; yanethdelgadomarrufo@gmail.com (E.D.-M.); lusmen.ruiz@gmail.com (L.R.-M.); fvalqui@unach.edu.pe (F.d.M.V.-P.); yajairagavidiap10@gmail.com (Y.G.-P.); 17090285@usp.br (G.B.-T.); blancacabrera11y@gmail.com (B.L.C.-C.); jose_marcelo@unj.edu.pe (J.L.M.-P.); k255040@dac.unicamp.br (K.L.A.-R.); 2 Departamento de Ciência e Tecnologia de Alimentos (LCA), Escola de Agricultura “Luiz de Queiroz”, Universidade de São Paulo, Piracicaba 13418-900, SP, Brazil; 3 Laboratorio de Dendrología, Facultad de Ingeniería, Universidad Nacional de Jaén (UNJ), Jaén 06801, Peru; 4 Department of Food Engineering, School of Food Engineering, University of Campinas, Monteiro Lobato Street, 80, Campinas 13083-862, SP, Brazil; 5 Grupo de Investigación en Tecnologías Emergentes Fotónicas (GITEF), Instituto de Investigación del Mejoramiento Productivo, Universidad Nacional Autónoma de Chota (UNACH), Chota 06121, Peru

**Keywords:** *Manihot esculenta*, starch, spontaneous fermentation, solar drying, gluten-free baking

## Abstract

Cassava (*Manihot esculenta*) is an important tropical crop and a valuable source of starch for food processing. This study evaluated the effect of three cassava ecotypes from Cajamarca, Peru-Puerto Huallape (PHE), Las Naranjas (LNE), and Puerto Chinchipe (PCE), on the quality and technological properties of naturally fermented sour cassava starch. Native starches were subjected to spontaneous fermentation followed by solar drying, and their fermentation kinetics, physicochemical, thermal, functional, and pasting properties were evaluated. The ecotypes required 4 to 5 days of fermentation to reach the target acidity and pH, followed by approximately 7 h of solar drying. Significant differences (*p* < 0.05) were observed among ecotypes in yield, pH, titratable acidity, moisture, water activity, whiteness index, expansion capacity, water absorption and solubility indices, gelatinization parameters and pasting properties. PHE exhibited the highest expansion capacity and peak viscosity, indicating greater potential for bakery applications; LNE showed the highest water absorption index, greater paste stability, and stronger molecular reassociation during cooling, whereas PCE exhibited greater fermentation intensity and a higher gelatinization temperature. Principal component analysis identified expansion capacity, whiteness, moisture, and water activity as the main variables differentiating the ecotypes. Furthermore, the results demonstrate significant differences among sour cassava starches obtained from three Peruvian ecotypes via natural fermentation and sun drying, allowing for their physicochemical differentiation; this provides a basis for selecting raw materials according to specific technological requirements, particularly for gluten-free baking applications.

## 1. Introduction

Cassava (*Manihot esculenta*) is one of the most important starch-rich crops worldwide and represents an essential source of energy for millions of people in tropical and subtropical regions [1,2]. Its importance has increased in the context of global food security due to its ability to withstand adverse environmental conditions, including prolonged drought and low-fertility soils, thereby providing advantages over other staple crops under climate change scenarios [3,4]. Furthermore, South America is considered one of the main centers of origin and genetic diversity of the species, harboring numerous ecotypes adapted to different agroecological conditions [5,6].

Starch is the main component of industrial interest in cassava roots, accounting for approximately 65–85% of the dry matter of the tuber [7,8]. Cassava starch also exhibits several advantageous sensory and technological characteristics, including a neutral flavor, high purity, digestibility, and good gelatinization capacity, which have led to its widespread use in the food, pharmaceutical, and biodegradable materials industries [9,10]. However, native cassava starch presents technological limitations associated with its thermal stability, rheological behavior, and resistance to industrial processing, creating a technological gap in meeting the emerging requirements for starch industrialization. Therefore, there is a growing need to develop physical, chemical, enzymatic, and biological modification strategies aimed at improving its functional properties [11,12,13].

In response to the limitations of native starch and the growing demand for new processing technologies, promising and cost-effective alternatives have been developed. Among these, natural fermentation combined with solar drying represents a traditional and sustainable approach for producing sour cassava starch. This ingredient is widely used in the food industry to produce gluten-free baked goods, which are highly demanded in countries such as Brazil, Colombia, and Paraguay [14,15]. During fermentation, the native microbiota, predominantly composed of lactic acid bacteria, produces organic acids and enzymes capable of partially modifying the granular structure of starch through the hydrolysis of amorphous regions [16,17]. Subsequently, solar drying may induce oxidation and photo-modification processes that contribute to further molecular changes, thereby promoting the development of distinctive functional properties that may be difficult to reproduce using conventional starch modification techniques [18,19].

Due to the physicochemical and structural modifications induced by these treatments, sour cassava starch acquires distinctive technological properties, including high expansion capacity, alterations in gelatinization temperature and pasting behavior, as well as improvements in sensory and textural attributes [20,21]. These techno-functional characteristics are particularly relevant to the formulation of gluten-free bakery products, as they contribute to increased specific volume and improved texture in products such as pão de queijo, pandebono, and chipa [22,23,24]. Consequently, sour cassava starch has emerged as a promising ingredient of scientific and industrial interest within the clean-label concept, particularly for the development of functional foods targeted at individuals with celiac disease or gluten sensitivity [14,25].

Furthermore, recent advances in starch science and technology have demonstrated that functional and technological properties are closely associated with structural factors, including granular morphology, amylose content, molecular organization, and degree of crystallinity [26,27]. In addition, the genetic origin of the plant material has been reported to play a determining role in the physicochemical characteristics of starch and its response to fermentation and drying processes [28,29]. Similarly, starches from different ecotypes within the same plant species have been shown to exhibit variations in chemical composition, hydration behavior, viscosity, thermal stability, and expansion capacity, which are highly relevant attributes for specialized food applications [26,30].

In particular, cassava fermentation has enabled the development of various functional food matrices through the reduction in cyanogenic glycosides and other antinutritional compounds, thereby improving the digestibility and antioxidant capacity of the final product [25,31,32]. However, most of these studies have focused on commercial cultivars from Africa, Brazil, and Asia. Consequently, the Andean-Amazonian ecotypes (Peru) remain poorly characterized, despite their adaptation to specific agroecological conditions, which makes them a promising source of starches with distinctive structural and functional characteristics [28,29]. Despite the socioeconomic importance of cassava in the Peruvian Amazon and the favorable conditions for its artisanal processing, sour cassava starch from local ecotypes remains uncharacterized. Specifically, no studies have evaluated how genetic variability among ecotypes influences the physicochemical, functional, and technological properties of sour cassava starch, thereby limiting the industrial utilization of these valuable agrobiological resources.

It was hypothesized that the genetic variability among Andean-Amazonian cassava ecotypes determines the chemical and structural characteristics of starch, thereby modulating its response to fermentation and solar drying and resulting in sour starches with distinct techno-functional profiles. To test this hypothesis, this study evaluated the influence of ecotype on the physical, chemical, hydration, pasting, and expansion properties relevant to bakery applications of artisanal sour cassava starch obtained from Manihot esculenta ecotypes from northern Peru. The findings provide a scientific basis for the valorization of local biodiversity through the development of sustainable functional ingredients.

## 2. Materials and Methods

**Figure 1:**
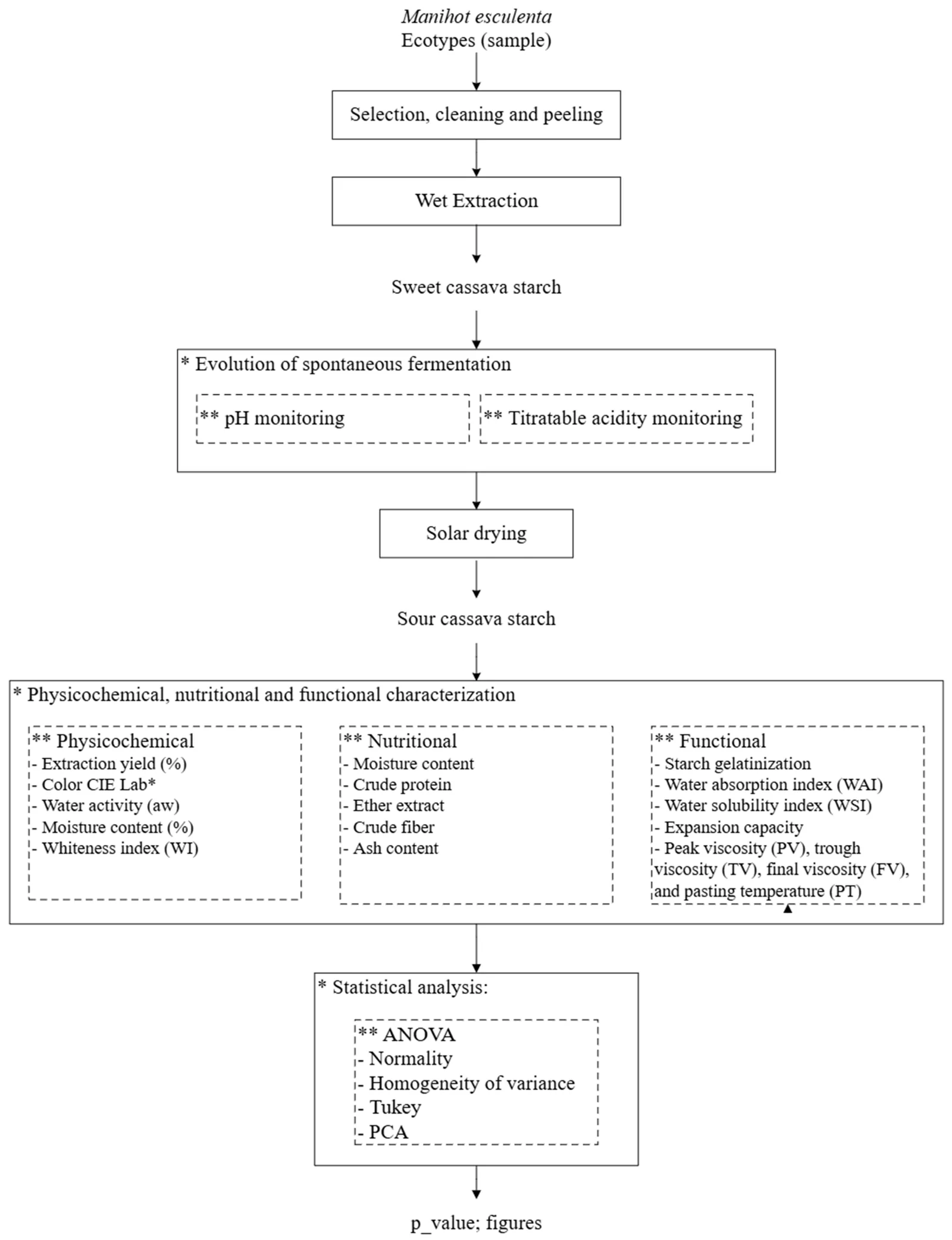
Experimental procedure for obtaining sour cassava starch.

The experimental procedure is shown in Figure 1. Likewise, each step of the flowchart is explained in the following paragraphs.

### 2.1. Cassava Root Sample

**Figure 2:**
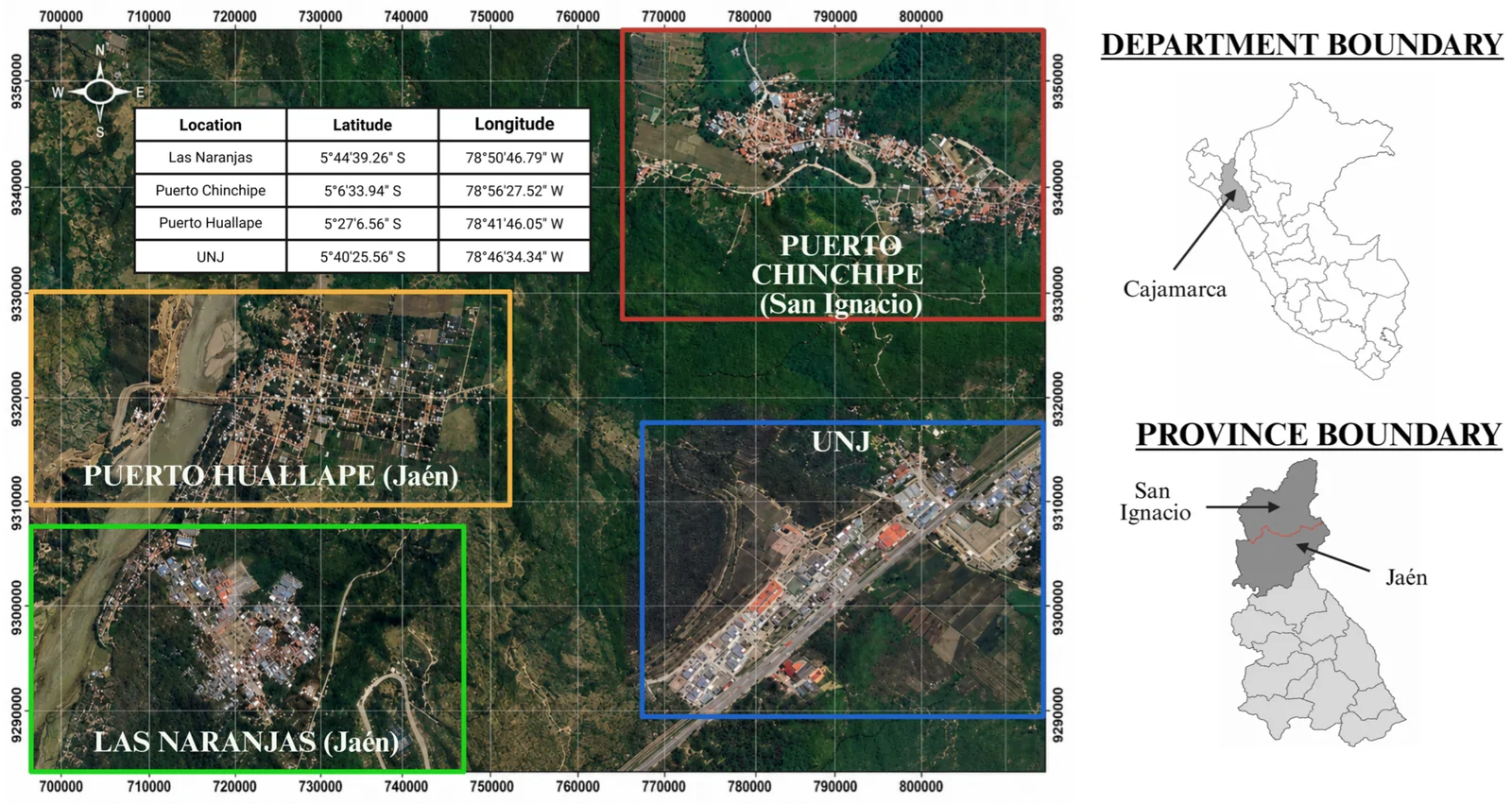
Geographic locations of the Manihot esculenta ecotype sampling sites. Note. Satellite imagery was obtained from Google Earth Pro (Google LLC, Mountain View, CA, USA, 2026).

Fresh cassava roots (*Manihot esculenta*) from three local ecotypes were collected from three cassava-producing areas in the Cajamarca region, Peru. The sampling sites were Puerto Huallape, Santa Rosa District, Jaén Province (5°27′06.56″ S, 78°41′46.05″ W; 664 m a,s,l,); Puerto Chinchipe, San José de Lourdes District, San Ignacio Province (5°06′33.94″ S, 78°56′27.52″ W; 1491 m a,s,l,); and Las Naranjas, Jaén District and Province (5°44′39.26″ S, 78°50′46.79″ W; 1194 m a,s,l,). These locations were selected because they represent cassava-producing areas with sufficient availability of plant material. The geographical locations and altitudes of the sampling sites are shown in Figure 2.

A sufficient quantity of fresh roots was collected from each location to obtain artisanal sour cassava starch. A total of 177 kg of cassava roots were collected, comprising 60 kg from the Puerto Huallape ecotype, 60 kg from the Puerto Chinchipe ecotype, and 57 kg from the Las Naranjas ecotype. Samples were selected using non-probability convenience sampling. Only roots free from mechanical and biological damage were selected, considering the availability of plant material, accessibility to the production sites, and the conditions required for collection, transportation, and subsequent experimental processing.

### 2.2. Wet Extraction of Cassava Starch

**Figure 3:**
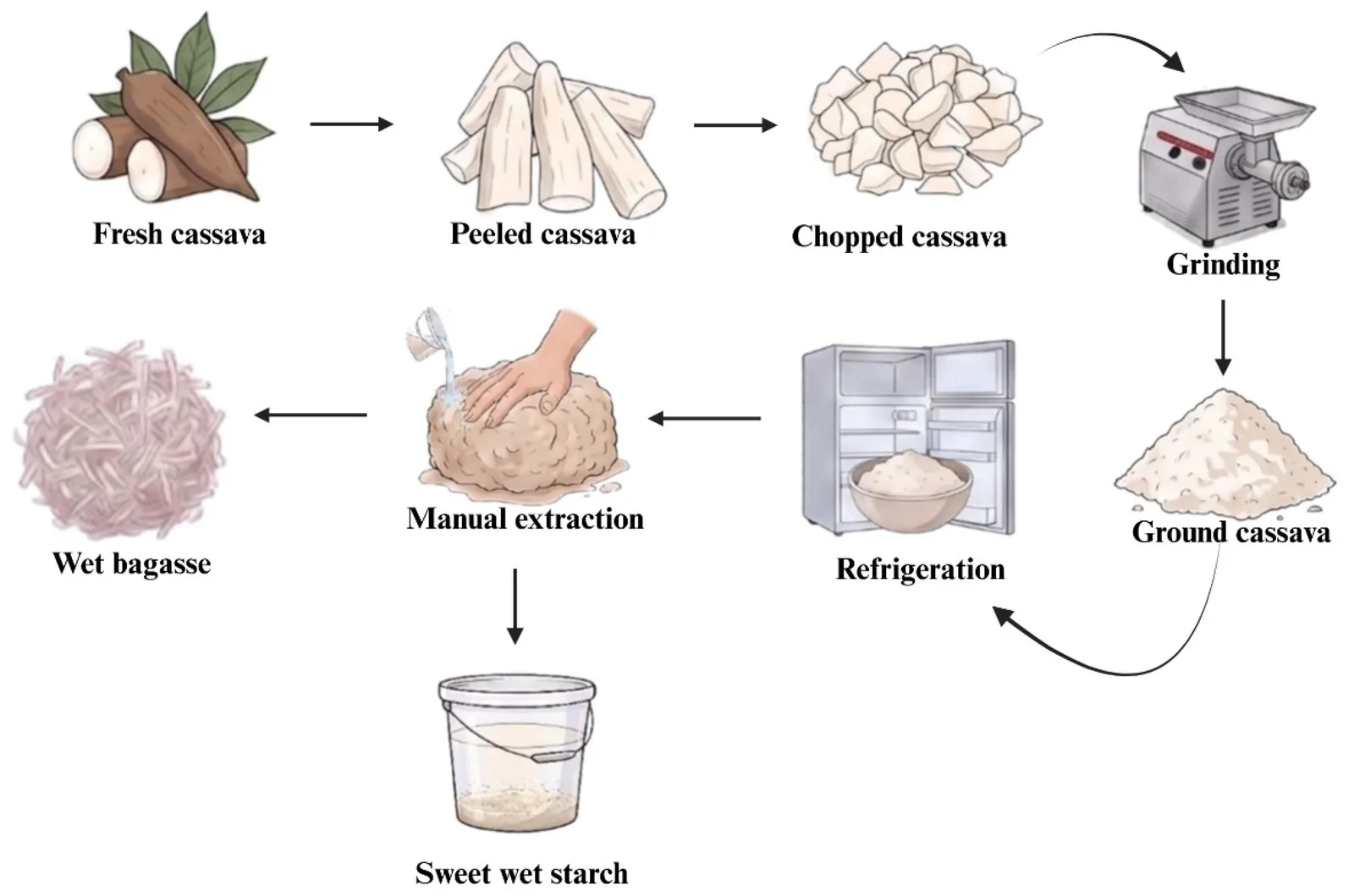
Process for obtaining wet sweet cassava starch from fresh Manihot esculenta roots.

**Figure 4:**
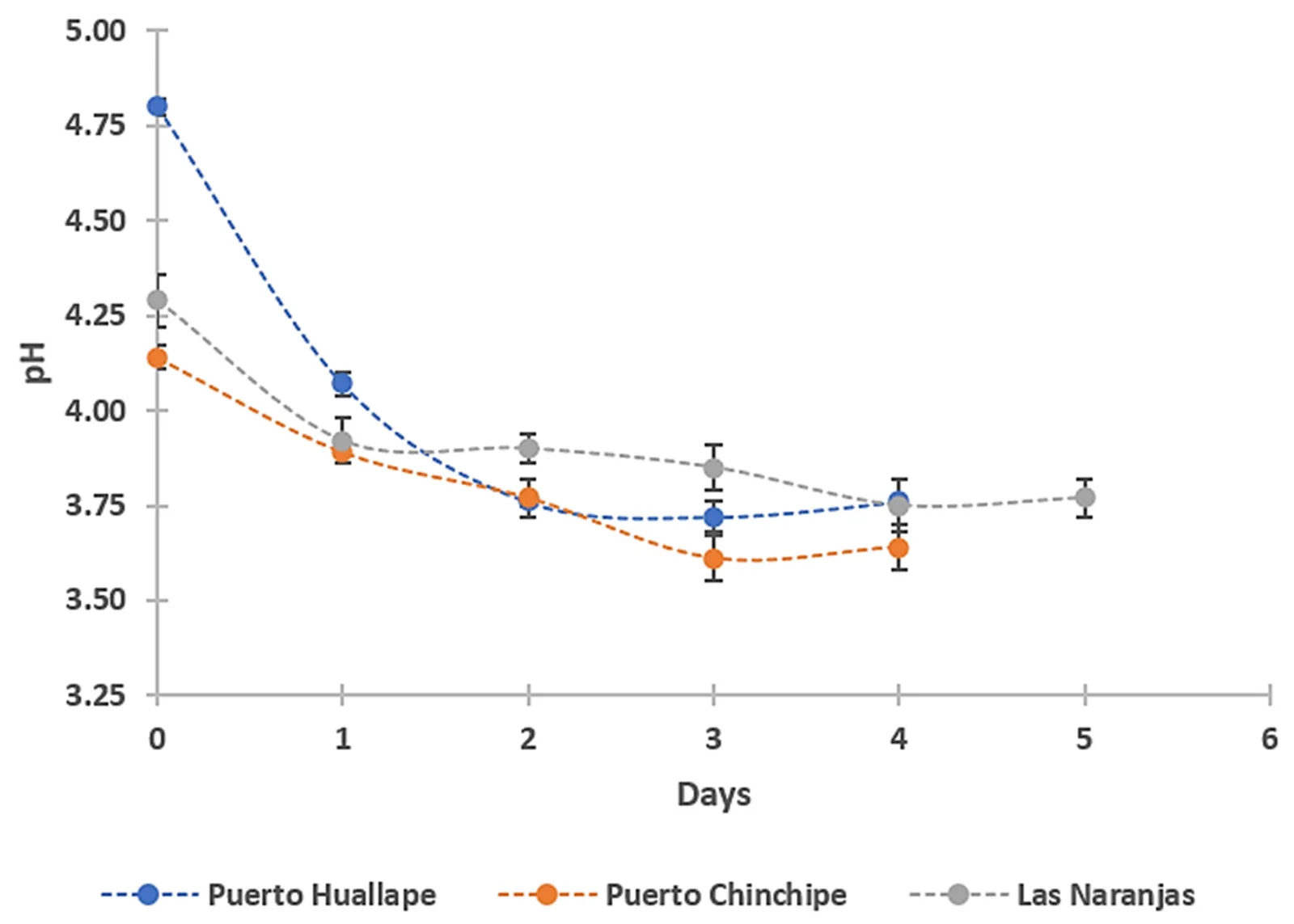
Variation in pH of the liquid phase during the spontaneous fermentation of starches from the Puerto Huallape (EPH), Puerto Chinchipe (EPC), and Las Naranjas (ELN) ecotypes. Values are presented as the mean ± standard deviation (n = 3).

Sweet cassava starch was obtained in the EPIFA-UNJ laboratory (Figure 3). Fresh roots from the three Manihot esculenta ecotypes were processed by wet extraction using a meat grinder (GRT-MC22, Blackbull, Lima, Peru). The resulting mash was maintained at 4 ± 1 °C for 24 h (CGD 580 L, Blackline, Lima, Peru) and subsequently subjected to five washing cycles (2w/5v). The starch fraction was recovered by sedimentation and decantation for 30 min. Finally, the resulting suspension was subjected to natural fermentation until a pH below 4.5 was reached (Figure 4).

### 2.3. Evolution of Spontaneous Fermentation

The fermentation process was monitored by determining the pH and titratable acidity of the medium. The pH was measured using a potentiometer (PRF60, APERA, Columbus, OH, USA) previously calibrated with pH 4.0 and 7.0 buffer solutions. For the analysis, a 10% (*w*/*v*) starch suspension was prepared, and the pH was measured directly in the suspension using the pH meter [33]. All measurements were performed in triplicate.

Titratable acidity was determined by alkalimetric titration. A suspension was prepared by mixing 1 g of sample with 9 g of distilled water and titrated with 0.1 N NaOH using phenolphthalein (three drops) as the indicator. Likewise, all measurements were performed in triplicate. The results were expressed as a percentage of lactic acid according to Equation (1).

(1) $$\mathrm{Titratable}\;\mathrm{acidity}\;\left(\%\mathrm{lactic}\;\mathrm{acid}\right)=\frac{V_{\mathrm{NaOH}}\times N_{\mathrm{NaOH}}\times 0.09\times 100}{m}$$

where V_NaOH_ is the volume of NaOH consumed during titration (mL), N_NaOH_ is the normality of the NaOH solution, 0.09 is the equivalent weight of lactic acid (g meq^−1^) and m is the mass of the analyzed sample (g).

### 2.4. Solar Drying

Wet sour cassava starch was subjected to solar drying on black polyethylene sheets protected with poliseda fabric at the facilities of the Universidad Nacional de Jaén (UNJ). Drying continued until the moisture content fell below 14% was achieved. Final moisture content (wet basis) was determined in triplicate according to the standardized AOAC 925.10 method [34].

### 2.5. Physicochemical and Nutritional Characterization

The physicochemical characterization of sour cassava starch included the determination of extraction yield, color, water activity (a_w_), and proximate composition. Moisture content (wet basis) was determined according to the standardized AOAC 925.10 method [34]. The extraction yield (%EY) was calculated as the percentage ratio between the mass of dry sour cassava starch obtained and the initial mass of processed cassava roots (Equation (2)).

Color parameters in the CIELAB space were measured using a PCE-CSM 8 spectrophotometer (PCE Instruments^®^, Meschede, Germany), configured with a D65 illuminant and a 10° standard observer, and expressed as L*, a*, and b* coordinates. The whiteness index (WI) was calculated from these coordinates according to Equation (3). Finally, water activity (aw) was measured using an AquaLab^®^ 4TE water activity meter (METER Group Inc., Pullman, WA, USA) based on the chilled-mirror dew-point technique.

(2) $$\%\mathrm{EY}=\frac{M_{a}}{M_{y\;}}\times 100$$

(3) WI=100−[100−L2+a2+b2]12

where *M_a_* = mass of sour starch obtained and *M_y_* = initial mass of cassava.

The proximate composition was determined using standardized methods established by AOAC International (2005) [35]. Specifically, moisture, crude protein, ether extract, crude fiber, and ash contents were determined according to AOAC methods 950.46, 984.13, 2003.05, 962.09, and 942.05, respectively, whereas total dietary fiber was quantified using the enzymatic-gravimetric AOAC 985.29 method. All analyses were performed in triplicate for each ecotype sample, and the results were expressed as g/100 g of sample on a wet basis.

### 2.6. Thermal Characterization

The thermal properties associated with starch gelatinization were determined by differential scanning calorimetry (DSC) using a DSC-60 Plus instrument (Shimadzu, Kyoto, Japan). Briefly, 4 mg of starch on a dry basis were weighed and mixed with 16 μL of distilled water at a starch-to-water ratio of 1:4 (*w*/*w*) in hermetically sealed aluminum pans. The samples were equilibrated at room temperature for 12 h and subsequently heated from 25 to 120 °C at a rate of 10 °C min^−1^, using an empty pan as the reference. The onset temperature (T_o_), peak temperature (T_p_), conclusion temperature (T_c_), and gelatinization enthalpy (ΔH) were determined from the resulting thermograms. Similarly, all measurements were performed in triplicate.

### 2.7. Functional Properties

The water absorption index (WAI) and water solubility index (WSI) were determined in triplicate according to the method described by Anderson et al. (1970) [36], with slight modifications. Briefly, 1.0 g of starch was dispersed in 12 mL of distilled water in 15 mL centrifuge tubes, and the suspension was thoroughly homogenized. The samples were incubated at 30 °C for 30 min in a water bath with periodic agitation and subsequently centrifuged at 3000 rpm for 10 min (TD5M, Bioridge, Shanghai, China). The supernatant was carefully separated from the sediment.

For WAI determination, the hydrated sediment was weighed immediately after centrifugation, and the results were expressed as grams of gel per gram of dry sample (Equation (4)). For WSI determination, the supernatant was transferred to previously weighed containers and dried at 100 °C for 24 h or until a constant weight was reached. The resulting residue corresponded to the soluble solids released from the starch, and the WSI was expressed as the percentage of soluble solids relative to the initial sample mass (Equation (5)). Similarly, all measurements were performed in triplicate.

(4) $$\mathrm{WAI}\left({\mathrm{gg}}^{-1}\right)=\frac{m_{g}}{m_{s}}$$

(5) $$\mathrm{WSI}\left(\%\right)=\frac{m_{\mathrm{ss}}}{m_{s}}\times 100$$

where m_s_ is the initial dry mass of the sample (g), mg is the mass of the hydrated sediment obtained after centrifugation (g), and m_ss_ is the mass of soluble solids recovered from the supernatant after drying to constant weight (g).

### 2.8. Expansion Capacity

Expansion capacity was evaluated based on specific volume (cm^3^ g^−1^) following the method described by Dos Santos Brito and Cereda (2015) [37], with modifications to the sample size and measurement conditions. For each treatment, three independent replicates of a cheese bread formulation containing sour starch (31%), oil (15%), water (15%), milk (15%), mozzarella cheese (15%), egg (8%) and salt (1%) were prepared. A 3.0 g portion of dough was weighed, individually molded, and baked at 200 °C for 20 min. After cooling to room temperature, the mass and volume of each baked unit were determined. Volume was measured using the displacement method with chia seeds. Expansion capacity was determined in triplicate for each replicate (n = 9), and specific volume (SV) was calculated as the ratio of the volume of the baked sample to its mass (Equation (6)).

(6) $$\mathrm{SV}({\mathrm{cm}}^{3}g^{-1})=\frac{V}{m}$$

where SV is the specific volume (cm^3^ g^−1^), V is the sample volume (cm^3^), and m is the sample mass (g).

### 2.9. Pasting Properties

The pasting properties of sour starches were determined in triplicate according to the AACC 76-21.01 method (AACC International, 2010) using a Rapid Visco Analyzer (RVA 4500, Perten Instruments, Macquarie Park, Australia). Briefly, 3.5 g of starch (dry basis) were weighed and dispersed in 25.0 g of distilled water in the instrument’s sample cup. The suspension was homogenized by applying ten vertical strokes with the stirring paddle before analysis. The temperature profile lasted 23 min and included controlled heating to 95 °C, followed by cooling to 50 °C. The peak viscosity (PV), trough viscosity (TV), final viscosity (FV), and pasting temperature (PT) were recorded from the resulting viscosity curves. Breakdown and setback were calculated as follows: Breakdown = peak viscosity − trough viscosity; Setback = final viscosity − trough viscosity. Likewise, all measurements were performed in triplicate.

### 2.10. Statistical Analysis

The effect of cassava ecotype on the evaluated properties was assessed by one-way ANOVA. Mean comparisons were performed using Tukey’s HSD test at a significance level of *p* < 0.05. All statistical analyses were conducted using Statgraphics Centurion XVIII (Statgraphics Technologies Inc., Warrenton, VA, USA), with complementary data processing in Microsoft Excel. Likewise, Principal component analysis (PCA) was performed on standardized data (mean = 0, SD = 1) using Jamovi software (version 2.7.41.).

## 3. Results

### 3.1. Spontaneous Fermentation of Sour Cassava Starch

**Figure 5:**
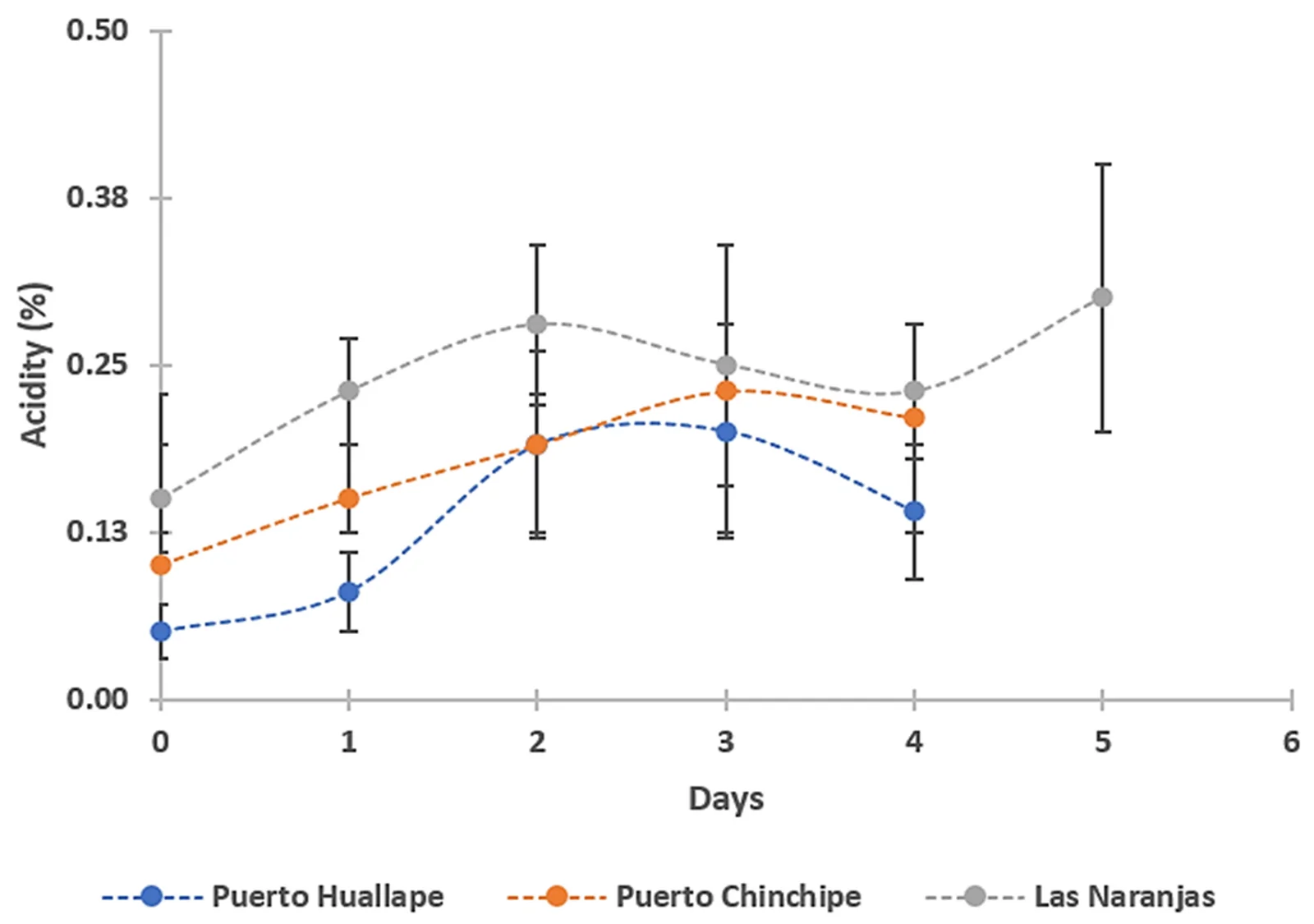
Changes in titratable acidity of the liquid phase during the spontaneous fermentation of starches from the Puerto Huallape (PHE), Puerto Chinchipe (PCE), and Las Naranjas (LNE) ecotypes. Values are presented as mean ± standard deviation (n = 3).

All three cassava ecotypes exhibited a progressive decrease in pH accompanied by an increase in titratable acidity during spontaneous fermentation (liquid phase) (Table 1; Figure 4 and Figure 5). At the beginning of fermentation, pH values ranged from 4.14 to 4.80, whereas acidity values ranged from 0.05 to 0.15%. As fermentation progressed, pH decreased to final values between 3.64 and 3.77, while titratable acidity increased.

Differences among ecotypes were observed in the rate and extent of acidification. PHE and PCE reached the final fermentation stage after four days, whereas LNE required five days. At the end of fermentation, LNE exhibited the highest titratable acidity, reaching 0.30 ± 0.10%, while the final pH values were 3.77 ± 0.05, 3.64 ± 0.06, and 3.75 ± 0.07 for PHE, PCE, and LNE, respectively (Table 1). During the fermentation period, the environmental temperatures recorded from the NASA POWER database ranged from 27.48 to 34.93 °C (Table 1).

The temporal evolution of pH showed a marked decrease during the first stages of fermentation, followed by stabilization toward the final fermentation stage (Figure 4). Conversely, titratable acidity progressively increased throughout fermentation, although the magnitude of the increase differed among ecotypes (Figure 5). LNE showed the highest final acidity, whereas PCE reached the lowest final pH.

### 3.2. Solar Drying and Moisture Reduction

The environmental conditions recorded during solar drying varied between the evaluation dates (Table 2). Temperature ranged from 14.28 to 33.55 °C, relative humidity (RH) from 30.89 to 62.34%, and wind speed from 1.20 to 1.80 m s^−1^. No precipitation was recorded during the drying evaluations.

After four hours of drying, moisture contents were 21.67 ± 1.31%, 19.43 ± 1.83%, and 17.57 ± 1.34% for EPH, EPC, and ELN, respectively (Table 3). After seven hours, the moisture contents decreased to 11.18 ± 1.43%, 9.11 ± 1.40%, and 7.63 ± 0.70%, respectively. The corresponding moisture reductions were 48.39%, 53.14%, and 56.55%.

LNE, dried on 12 November 2024, exhibited the lowest final moisture content and the greatest moisture reduction. Approximately seven hours of solar exposure were sufficient to obtain moisture contents below 12% for all three ecotypes under the evaluated conditions.

### 3.3. Physicochemical Properties and Proximate Composition

Significant differences among ecotypes were observed for yield, moisture content, pH, titratable acidity, whiteness index, water activity, and dough expansion (*p* < 0.05; Table 4). PHE showed the highest yield (12.92 ± 1.08%) and moisture content (11.06 ± 0.60%), whereas LNE showed the lowest values for both parameters, with 11.20 ± 0.28% yield and 7.42 ± 0.53% moisture.

The final pH values were 5.64 ± 0.51, 4.17 ± 0.10, and 4.28 ± 0.13 for PHE, PCE, and LNE, respectively. Titratable acidity was highest in PCE (0.19 ± 0.01%), followed by LNE (0.16 ± 0.01%) and PHE (0.08 ± 0.01%). The whiteness index ranged from 91.71 ± 0.10 for LNE to 93.12 ± 0.75 for PHE, while water activity ranged from 0.38 ± 0.04 to 0.54 ± 0.04.

Dough expansion differed significantly among ecotypes. PHE showed the highest expansion index (2.37 ± 0.13 cm^3^ g^−1^), followed by PCE (2.18 ± 0.17 cm^3^ g^−1^) and LNE (1.92 ± 0.03 cm^3^ g^−1^).

The proximate composition also showed differences among samples (Table 5). Nitrogen-free extract represented the major fraction, ranging from 88.67 to 91.18%. Total dietary fiber ranged from 1.10% in LNE to 2.30% in PCE. Protein content ranged from 0.48 to 0.66%, either extracted ranged from 0.00 to 0.13%, crude fiber from 0.00 to 0.08%, and ash from 0.10 to 0.28%.

### 3.4. Principal Component Analysis

**Figure 6:**
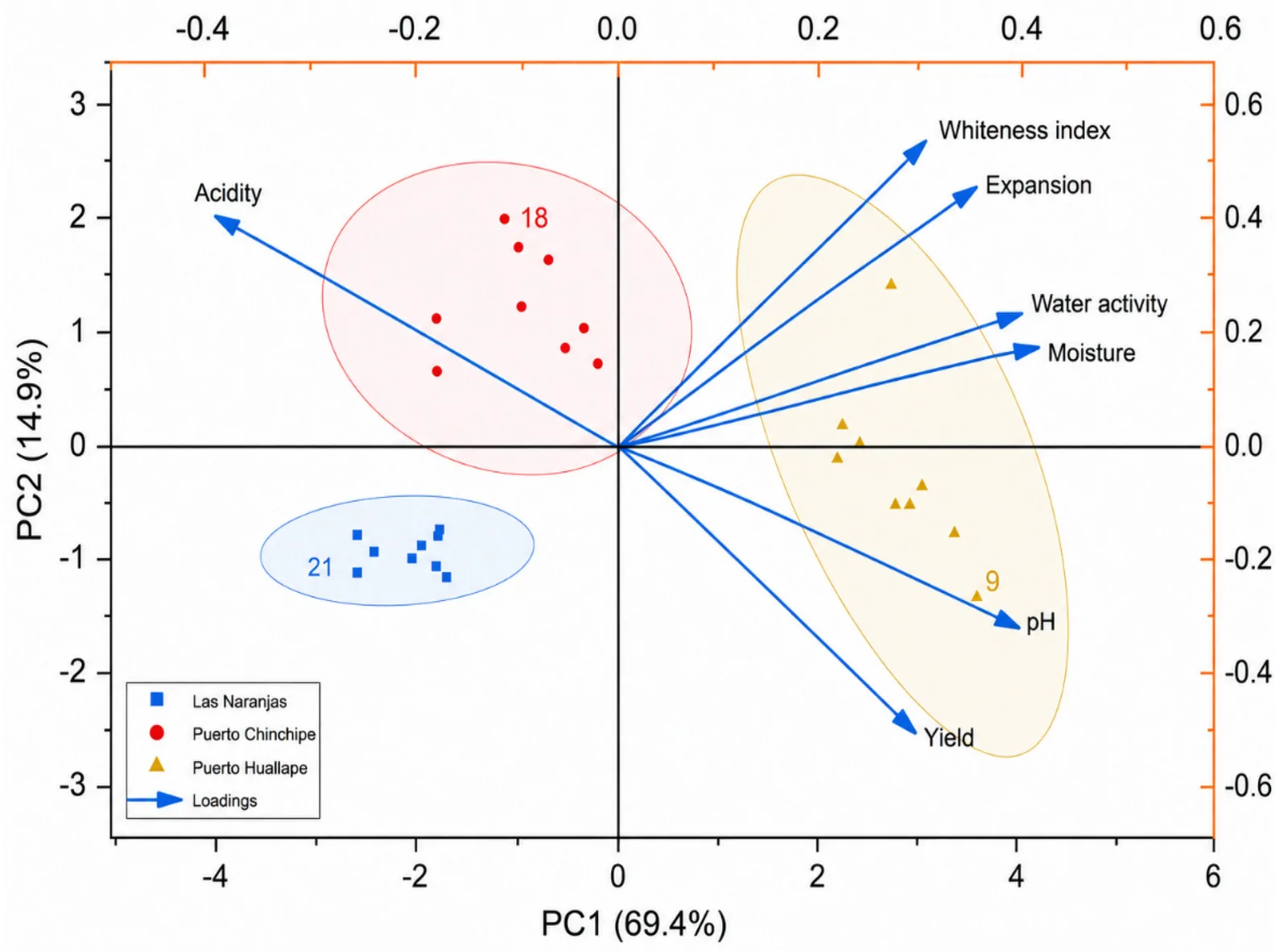
Biplot of the Principal Component Analysis of the physicochemical and quality properties of sour starches from three ecotypes.

The first two principal components, PC1 and PC2, explained 84.3% of the total variance, accounting for 69.4% and 14.9%, respectively (Figure 6). PC1 showed positive associations with expansion index, whiteness index, water activity, and moisture content, whereas acidity was negatively associated with this component.

PHE was located predominantly on the positive side of PC1 and was associated with expansion index, whiteness, moisture content, and water activity. LNE was positioned toward the negative side of PC1 and showed an opposite relationship with these variables.

PC2 was mainly associated with acidity in the positive direction, whereas pH and yield showed important negative associations. PCE was positioned predominantly in the positive region of PC2 and close to the acidity vector.

### 3.5. Thermal Properties

Significant differences were observed in the thermal properties of the sour starches (*p* < 0.05; Table 6). PHE showed onset, conclusion, and peak temperatures of 62.96 ± 1.74, 79.30 ± 1.14, and 70.87 ± 0.61 °C, respectively, with an enthalpy of 9.15 ± 1.81 J g^−1^.

PCE exhibited a peak temperature of 71.72 ± 0.44 °C and an enthalpy of 8.12 ± 0.77 J g^−1^. ELN showed the lowest onset temperature (53.30 ± 2.91 °C), conclusion temperature (72.63 ± 2.52 °C), peak temperature (64.13 ± 0.37 °C), and enthalpy (8.03 ± 1.72 J g^−1^).

The thermal profile therefore differed among ecotypes, particularly for LNE, which exhibited lower transition temperatures and enthalpy than PHE and PCE.

### 3.6. Functional Properties

Significant differences were observed in water solubility index (WSI) and water absorption index (WAI) among ecotypes (*p* ≤ 0.05; Table 7). PHE exhibited the highest WSI (1.02 ± 0.04%), followed by PCE (0.63 ± 0.03%) and LNE (0.16 ± 0.01%).

In contrast, LNE exhibited the highest WAI (1.98 ± 0.04 g g^−1^), followed by PCE (1.85 ± 0.04 g g^−1^) and PHE (1.79 ± 0.03 g g^−1^). Thus, PHE showed the highest solubilization capacity, whereas LNE exhibited the greatest water absorption capacity.

### 3.7. Pasting Properties

**Figure 7:**
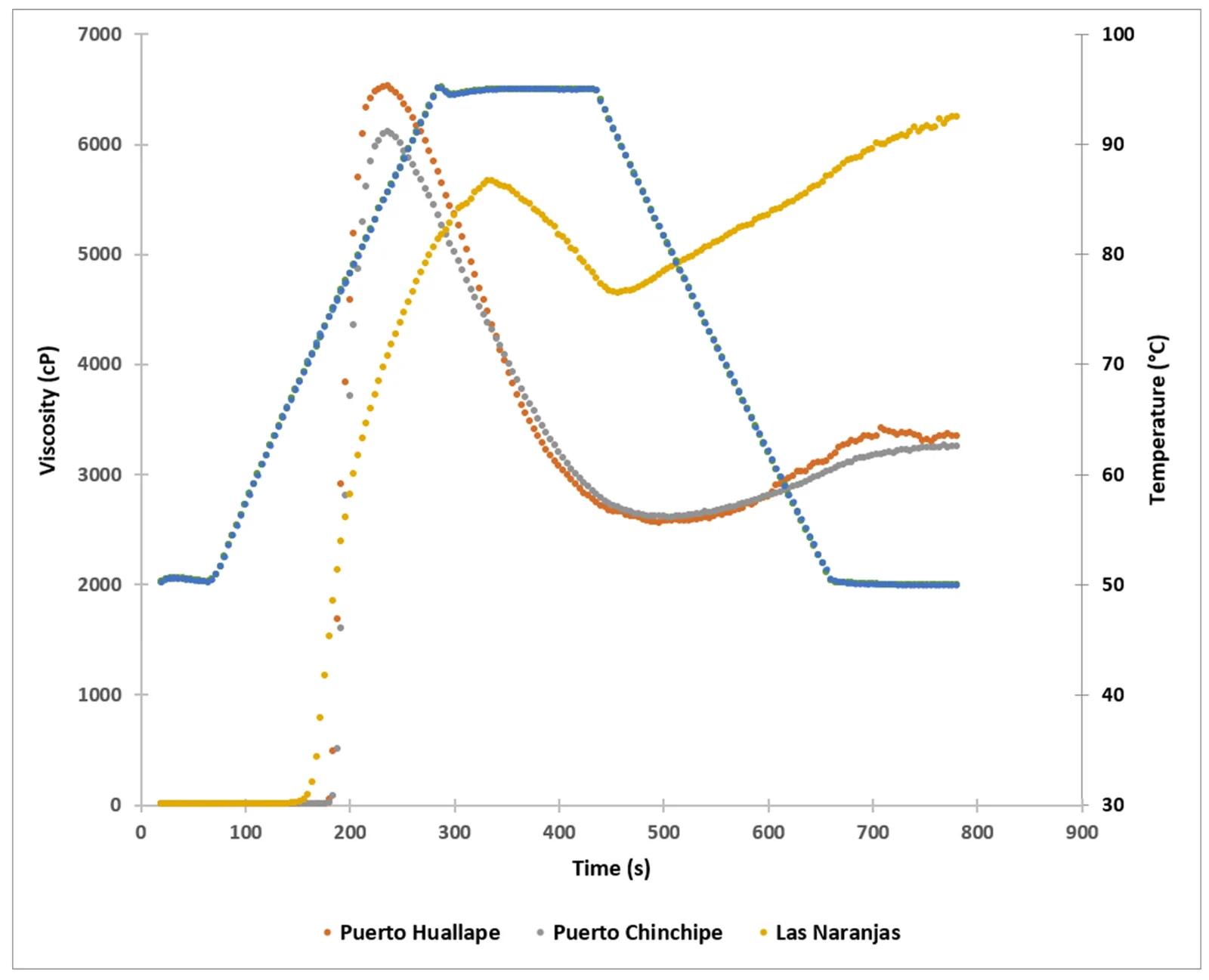
Pasting curves of sour starches obtained from different cassava ecotypes.

Significant differences were observed among the three ecotypes for the pasting parameters (Table 8; Figure 7). PHE exhibited the highest peak viscosity (6502 ± 58.81 cP), followed by PCE (6131 ± 86.26 cP) and LNE (5671 ± 62.22 cP). The minimum viscosity was highest for LNE (4645 ± 14.45 cP), whereas PHE and PCE showed lower values of 2543 ± 47.57 and 2606.3 ± 56.36 cP, respectively. Final viscosity was also highest for LNE (6248.67 ± 206.95 cP), compared with 3354 ± 173.68 cP for PHE and 3257 ± 79.22 cP for PCE.

The pasting temperature was significantly lower for LNE (69.62 ± 0.51 °C) than for PHE (74.65 ± 0.48 °C) and PCE (74.83 ± 0.46 °C). LNE also showed the lowest breakdown (1026 cP) and the highest setback (1604 cP), whereas PHE and PCE showed breakdown values of 3961 and 3525 cP and setback values of 819 and 651 cP, respectively. The pasting curves (Figure 7) showed a pronounced decrease in viscosity after the peak for PHE and PCE, whereas LNE exhibited a smaller decrease followed by a greater increase during cooling.

## 4. Discussion

### 4.1. Ecotype-Dependent Fermentation Behavior

The progressive decrease in pH and simultaneous increase in titratable acidity of the liquid phase observed in all three ecotypes are consistent with the characteristic acidification pattern of spontaneously fermented cassava starch. This behavior is generally associated with the metabolic activity of lactic acid bacteria and other fermentative microorganisms capable of converting available carbohydrates into organic acids [14,38]. The final pH values of the liquid phase observed in the present study were within the ranges previously reported for naturally fermented sour cassava starches [31,39].

Despite the common acidification pattern, the differences in fermentation duration and final acidity among ecotypes suggest that the raw material may influence fermentation kinetics. PHE and PCE reached the final fermentation condition after four days, whereas LNE required five days and reached the highest final titratable acidity. Previous studies have shown that differences in cassava genotype can be associated with variations in starch composition and structural characteristics, which may affect substrate accessibility and, consequently, the development and activity of the indigenous fermentative microbiota [28,30,40].

The slower acidification pattern observed for LNE, followed by greater accumulation of titratable acidity may also reflect differences in microbial succession. During spontaneous fermentation, the composition of the indigenous microbiota and the succession of microbial populations can influence the rate of organic acid production and the evolution of pH [38,41]. However, because the microbial community was not characterized in the present study, this interpretation should be regarded as a plausible explanation rather than direct evidence of a microbial mechanism.

The environmental temperature recorded during fermentation remained within a range compatible with the development of lactic acid bacteria [14]. Nevertheless, the observed temperature variation indicates that environmental conditions were not completely constant. Therefore, the differences among ecotypes cannot be attributed exclusively to genotypes. A more definitive assessment of the ecotype effect would require controlled fermentation conditions and, ideally, characterization of the microbial communities associated with each material.

### 4.2. Effect of Solar Drying Conditions on Moisture Reduction

Solar drying produced a substantial reduction in moisture within approximately seven hours; however, the drying behavior should be interpreted in relation to the meteorological conditions prevailing on each evaluation date. LNE reached the lowest final moisture content, coinciding with the highest maximum temperature, lowest relative humidity, and higher wind speed recorded during the drying period. These conditions favor moisture transfer by increasing the vapor pressure gradient between the product and surrounding air and by enhancing convective mass transfer at the product surface.

The greater moisture reduction observed for LNE is therefore compatible with the environmental conditions recorded during its drying period. Similar relationships between solar-drying performance and temperature, relative humidity, and air circulation have been reported for fermented cassava starch [30,42]. Nevertheless, an important limitation of the present comparison is that the ecotypes were not dried simultaneously under identical meteorological conditions. Consequently, the observed differences in final moisture cannot be interpreted exclusively as an ecotype effect.

The final moisture content obtained for LNE was below 10%, indicating that the solar drying procedure was capable of producing a relatively low-moisture material under favorable environmental conditions. Lower moisture and water availability can contribute to improved microbiological stability during storage [14]. However, storage stability itself was not evaluated in this study; therefore, the present results demonstrate the attainment of a low moisture content rather than directly demonstrating extended shelf life.

The approximately seven-hour drying period should consequently be regarded as an empirical reference under the environmental conditions evaluated rather than as a universal processing time. Solar drying is inherently dependent on meteorological variability, and differences in radiation, temperature, relative humidity, and air movement can substantially modify drying kinetics. In addition, intrinsic characteristics of the starch, including initial moisture content and particle structure, may contribute to differences in water removal [18,43].

### 4.3. Physicochemical Characteristics and Expansion Capacity

The physicochemical and functional properties observed in this study generally fell within the ranges reported for cassava sour starch; however, some differences were identified in gelatinization temperature, water absorption index (WAI), water solubility index (WSI), viscosity, and specific volume. These variations may be associated with the relatively short fermentation period and the drying conditions, which likely limited the extent of starch modification compared with traditional sour starch production processes involving longer fermentation and sun-drying. Therefore, the results should be interpreted within the context of the processing conditions applied, highlighting the importance of fermentation and drying parameters in determining the final functional characteristics of cassava sour starch [30,43].

The significant differences in physicochemical properties indicate that the three ecotypes did not exhibit identical responses to the fermentation and drying process. The most relevant technological difference was the expansion index, with PHE exhibiting the highest value, followed by PCE and LNE. Expansion is a key functional attribute of sour cassava starch because of its relationship with the volume and textural characteristics of baked products, particularly gluten-free formulations [30,43].

The higher expansion capacity of PHE, despite its lower final acidity than PCE, indicates that expansion cannot be explained solely by the degree of acidification. Fermentation can induce several simultaneous structural modifications in starch, including changes in molecular organization and the physicochemical environment of starch granules. Subsequent drying may further modify these characteristics, ultimately affecting the ability of sour starch to develop gas-retaining structures during baking [18,30].

The comparison among PHE, PCE, and LNE further supports this interpretation. PCE exhibited the highest acidity but an intermediate expansion index, whereas LNE showed the lowest moisture and water activity but also the lowest expansion. Thus, neither acidity nor dehydration alone adequately explains the expansion behavior observed in the present study.

These results suggest that the technological functionality of sour cassava starch is governed by the interaction of several structural and physicochemical factors generated throughout processing. In this context, the ecotype may influence the initial starch structure and composition, whereas fermentation and drying may modify these characteristics. Because the present study did not directly characterize starch molecular structure, amylose/amylopectin organization, or granule morphology, these mechanisms should be considered interpretative rather than directly demonstrated.

### 4.4. Proximate Composition and Its Relationship with Functionality

The high nitrogen-free extract content observed for all samples is consistent with the carbohydrate-rich nature of cassava starch [8]. The differences in dietary fiber content, particularly the higher value observed for PCE, provide evidence of compositional variability among the evaluated materials.

However, the proximate composition alone did not explain the differences in expansion capacity. PCE exhibited the highest dietary fiber content but did not show the highest expansion, whereas PHE presented the greatest expansion despite having an intermediate fiber content. This observation indicates that the technological performance of sour starch is not determined by a single compositional parameter.

Previous studies have associated the functionality of sour cassava starch with structural and molecular modifications occurring during fermentation and drying rather than exclusively with the initial proximate composition [18,30,43]. The present results are compatible with this interpretation, although direct structural analyses would be necessary to establish the molecular basis of the observed differences.

### 4.5. Multivariate Differentiation of the Ecotypes

The PCA provided an integrated view of the relationships among physicochemical and functional variables. The first two principal components accounted for 84.3% of the total variance, indicating that most of the variation captured by the analyzed variables could be represented in a two-dimensional space.

The positive association of PC1 with expansion, whiteness, moisture content, and water activity was consistent with the positioning of PHE, which exhibited the highest expansion index and comparatively higher moisture and water activity. Conversely, LNE was positioned toward the negative side of PC1 and showed lower values for moisture, water activity, and expansion.

PC2 was mainly associated with acidity, with PCE positioned closer to the acidity vector. This pattern agrees with the higher final acidity observed for PCE in the physicochemical characterization. Thus, the PCA supports the existence of distinct physicochemical profiles among the ecotypes.

Importantly, PCA identifies associations among variables but does not establish causality. The observed separation may reflect the combined effects of genotype, fermentation kinetics, drying conditions, and their interactions. In particular, because the ecotypes were subjected to solar drying under different environmental conditions, the PCA should be interpreted as a description of the overall physicochemical differentiation of the resulting sour starches rather than as evidence that genotype alone caused the observed separation.

### 4.6. Thermal Behavior and Structural Implications

The observed differences in thermal properties should be interpreted with caution. Although they may be associated with intrinsic characteristics of the cassava materials, fermentation duration and drying conditions also differed among samples. LNE underwent a longer fermentation period and was dried under meteorological conditions different from those experienced by PHE and PCE. Previous studies have shown that both fermentation intensity and drying conditions can significantly modify starch thermal behavior. Therefore, the differences in gelatinization parameters cannot be attributed exclusively to ecotype-related factors [43].

The higher peak temperature observed for PCE, despite its relatively high acidity, further indicates that thermal behavior cannot be directly inferred from fermentation acidity alone. Conversely, LNE exhibited both the greatest moisture reduction during solar drying and the lowest thermal transition temperatures and enthalpy. This pattern is compatible with the possibility that the combination of fermentation and drying conditions altered the molecular organization of LNE more extensively.

The higher enthalpy observed for PHE was also accompanied by the highest expansion index. This parallel behavior suggests an association between the thermal characteristics of the starch and its subsequent technological performance. Nevertheless, ΔH represents the energy required for the gelatinization transition and should not be interpreted independently as a direct measure of expansion capacity. Structural characterization is required to establish the mechanisms underlying the association.

Overall, the thermal results support the interpretation that fermentation and drying generated differences in starch organization among the ecotypes, consistent with previous reports on fermented cassava starches [18,30,43].

### 4.7. Water Interaction Properties

The contrasting WSI and WAI values observed among the ecotypes indicate differences in starch–water interactions. PHE showed the highest WSI, whereas LNE exhibited the highest WAI. Higher solubility can be associated with greater disruption or modification of starch structures during processing, facilitating the release of soluble components into the aqueous phase [14,39].

Conversely, the greater WAI observed for LNE indicates a higher capacity to retain or absorb water under the conditions of the assay. Differences in starch molecular organization and granular characteristics may contribute to this behavior [8]. Genotypic variability has previously been associated with differences in cassava starch structure and functionality [9,28].

The contrasting WSI and WAI profiles also reinforce the multidimensional nature of sour starch functionality. A material with high solubility does not necessarily exhibit the highest water absorption, indicating that these parameters capture different aspects of starch–water interaction.

### 4.8. Pasting Behavior and Technological Implications

The pasting profiles revealed pronounced differences among ecotypes, particularly between LNE and the other two starches. PHE and PCE exhibited higher peak viscosities, indicating greater viscosity development during heating and gelatinization. Differences in peak viscosity among cassava genotypes have been associated with variations in starch molecular organization, amylose and amylopectin characteristics, and granule swelling behavior [9,28,40].

In contrast, LNE showed a markedly higher trough viscosity and the lowest breakdown value. Breakdown reflects the loss of viscosity associated with the combined effects of heating and shear after the viscosity peak. Therefore, the lower breakdown observed for LNE indicates greater resistance of the hot paste to these conditions. This interpretation is consistent with the smaller decrease in viscosity observed after the peak in Figure 7.

LNE also showed the highest final viscosity and setback value. The greater increase in viscosity during cooling indicates a stronger tendency for molecular reassociation after gelatinization. Setback is commonly associated with the reassociation of starch chains during cooling and may contribute to firmer gel formation and retrogradation [36,40].

The lower pasting temperature observed for LNE indicates that less thermal energy was required to initiate the development of viscosity under the RVA conditions. Differences in pasting temperature and viscosity parameters among cassava starches have been attributed to varietal characteristics as well as structural changes induced during fermentation and drying [14,16,30].

Taken together, the pasting results demonstrate that the three ecotypes provide markedly different rheological profiles. PHE and PCE exhibited greater peak viscosity development, whereas LNE showed greater resistance to breakdown, higher final viscosity, and stronger setback. These contrasting profiles may be relevant for different food applications, although application-specific performance would require validation in the corresponding food matrix.

### 4.9. Integrated Interpretation of Ecotype Effects

Although significant differences were observed among the sour cassava starches produced from the three evaluated ecotypes, these differences cannot be attributed exclusively to genetic variation. The observed variability may have resulted from the combined influence of multiple factors, including genotype, agroecological conditions, soil characteristics, crop management practices, root maturity, fermentation dynamics, and solar drying conditions [30]. Previous studies have demonstrated that starch susceptibility to physicochemical modifications during fermentation and drying is influenced not only by genetic background but also by environmental and post-harvest factors. Therefore, the differences identified in the present study should be interpreted as the outcome of the interaction between plant material and processing conditions rather than as an isolated ecotype effect. In addition, fermentation was conducted under artisanal conditions, and the drying process was carried out under naturally fluctuating environmental conditions. Consequently, part of the variability observed in thermal, rheological, and functional properties may reflect differences in fermentation duration, microbial activity, and meteorological conditions during drying.

Taken together, the results showed clear differences among the sour cassava starches obtained from the three evaluated Manihot esculenta ecotypes in terms of physicochemical, thermal, hydration, and pasting properties. PHE was characterized by greater expansion and WSI, PCE by higher acidity and thermal peak temperature, and LNE by lower moisture and water activity, greater WAI, lower thermal transition temperatures, and higher final and setback viscosities.

These differences are compatible with the hypothesis that the characteristics of the cassava ecotype contribute to the properties of the resulting sour starch. However, the experimental design also included uncontrolled or partially controlled process variables, particularly during spontaneous fermentation and solar drying. These differences are compatible with the hypothesis that intrinsic characteristics associated with cassava ecotype identity may contribute to the properties of the resulting sour starch.

This distinction is particularly important for the solar-drying stage, because LNE was processed under more favorable meteorological conditions than PHE and PCE. Future studies should therefore evaluate the ecotypes under standardized drying conditions and controlled fermentation, complemented by starch structural characterization and microbial community analysis. Such an approach would allow the relative contributions of genotype, fermentation, and drying to be disentangled more rigorously.

Despite this limitation, the combined physicochemical, thermal, functional, and pasting data provide evidence that spontaneous fermentation followed by solar drying can generate sour cassava starches with differentiated technological properties. The distinct profiles observed among PHE, PCE, and LNE may provide preliminary information for the selection of cassava materials in starch-based food applications requiring specific functional attributes.

## 5. Conclusions

Significant differences were observed among the sour cassava starches obtained from the three evaluated cassava materials under artisanal fermentation and solar drying conditions. These differences likely reflect the combined influence of plant material characteristics, fermentation dynamics, and environmental conditions during drying. Consequently, the present study does not allow the specific contribution of genetic factors to be distinguished from processing-related effects. Although all three ecotypes followed the typical acidification pattern associated with natural fermentation, differences were observed in fermentation kinetics and in the intensity of the transformations induced during processing.

The interaction between fermentation and sun drying generated distinct responses among the ecotypes, as evidenced by variations in expansion capacity, thermal properties, water interactions, and pasting profiles. The Puerto Huallape ecotype stood out for its highest expansion capacity and positive association with technological attributes of interest for gluten-free baking applications, whereas Las Naranjas exhibited greater structural stability and a stronger tendency toward retrogradation. In turn, Puerto Chinchipe was characterized by more intense fermentation and intermediate technological performance. From an industrial perspective, PHE could be particularly suitable for gluten-free bakery products requiring high expansion and volume development, such as cheese bread and specialty baked goods. LNE may be advantageous in formulations requiring greater paste stability, water retention, and resistance to breakdown during thermal processing, whereas PCE may represent an intermediate alternative combining fermentation intensity with balanced technological properties. From an industrial perspective, PHE could be particularly suitable for gluten-free bakery products requiring high expansion and volume development, such as cheese breads and specialty baked goods. LNE may be advantageous in formulations requiring greater paste stability, water retention, and resistance to breakdown during thermal processing, whereas PCE may represent an intermediate alternative combining fermentation intensity with balanced technological properties.

Nevertheless, the interpretation of ecotype-related differences should consider that solar drying was conducted under naturally fluctuating environmental conditions. Therefore, part of the observed variability may reflect the combined influence of ecotype and meteorological factors during drying.

Multivariate analysis confirmed that sour starch quality does not depend on a single parameter, but rather on the complex interaction among genetic, microbiological, and technological factors that modify starch structural organization during processing. Overall, these findings expand current knowledge of the influence of ecotypic origin on cassava sour starch production and provide scientific criteria for selecting raw materials aimed at developing ingredients with specific functional properties for the food industry.

## Figures and Tables

**Table 1:** Physicochemical parameters recorded for the liquid phase during fermentation of starches from the EPH, EPC, and ELN ecotypes (mean ± SD, n = 3).

Date (2024)	Tmax (°C)	Tmin (°C)	pH			Acidity (%)		
			PHE	PCE	LNE	PHE	PCE	LNE
06/11	33.95	19.27	4.80 ± 0.02 ^a^	4.14 ± 0.03 ^b^	4.29 ± 0.07 ^c^	0.05 ± 0.02 ^a^	0.10 ± 0.01 ^ab^	0.15 ± 0.04 ^b^
07/11	31.15	18.45	4.07 ± 0.03 ^a^	3.89 ± 0.01 ^b^	3.92 ± 0.06 ^b^	0.08 ± 0.03 ^a^	0.15 ± 0.02 ^b^	0.23 ± 0.04 ^c^
08/11	34.93	15.40	3.76 ± 0.01 ^a^	3.77 ± 0.05 ^b^	3.90 ± 0.04 ^b^	0.19 ± 0.07 ^a^	0.19 ± 0.04 ^a^	0.28 ± 0.06 ^a^
09/11	32.80	19.46	3.72 ± 0.04 ^a^	3.61 ± 0.06 ^b^	3.85 ± 0.06 ^c^	0.20 ± 0.08 ^a^	0.23 ± 0.07 ^a^	0.25 ± 0.09 ^a^
10/11	27.48	20.16	3.76 ± 0.06 ^a^	3.64 ± 0.06 ^a^	3.75 ± 0.07 ^a^	0.14 ± 0.05 ^a^	0.21 ± 0.07 ^a^	0.23 ± 0.05 ^a^
11/11	34.91	17.42	-	-	3.77 ± 0.05 ^a^	-	-	0.30 ± 0.10 ^a^

Note. Maximum and minimum temperatures were obtained from the NASA POWER database (https://power.larc.nasa.gov/data-access-viewer/, accessed on 15 November 2024). PHE: Puerto Huallape Ecotype, PCE: Puerto Chinchipe Ecotype, LNE: Las Naranjas Ecotype. Different lowercase letters within the same row and for the same parameter indicate significant differences between ecotypes according to Tukey’s test (*p* ≤ 0.05).

**Table 2:** Environmental conditions at EPIFA-UNJ during solar drying of sour starch.

Date (2024)	Tmax (°C)	Tmin (°C)	RH (%)	Precipitation	Solar Irradiance (MJ/m^2^/day)	Wind Speed (WS2M)
10/11	27.48	20.16	62.34	0.00	24.02	1.20
12/11	33.55	14.28	30.89	0.00	20.34	1.80

Note. NASA POWER database (https://power.larc.nasa.gov/data-access-viewer/, accessed on 15 November 2024), RH: relative humidity.

**Table 3:** Moisture content of sour starch during solar drying under environmental conditions at EPIFA-UNJ (mean ± SD, n = 3).

Ecotype	Date (2024)	Moisture at 4 h (%)	Moisture at 7 h (%)	Moisture Reduction (%)
PHE	10/11	21.67 ± 1.31 ^a^	11.18 ± 1.43 ^a^	48.39
PCE	10/11	19.43 ± 1.83 ^ab^	9.11 ± 1.40 ^ab^	53.14
LNE	12/11	17.57 ± 1.34 ^a^	7.63 ± 0.70 ^b^	56.55

Note. Total reduction (%) = [(Moisture at 4 h − Moisture at 7 h)/Moisture at 4 h] × 100, PHE: Puerto Huallape Ecotype, PCE: Puerto Chinchipe Ecotype, LNE: Las Naranjas Ecotype. Different lowercase letters within the same row and for the same parameter indicate significant differences between ecotypes according to Tukey’s test (*p* ≤ 0.05).

**Table 4:** Physicochemical properties of sour starch obtained from cassava ecotypes (MEAN ± SD, n = 3).

Sour Starch	Yield (%)	Moisture (%)	pH	Acidity (%)	Whiteness Index	Water Activity (a_w_)	Dough Expansion (cm^3^ g^–1^)
PHE	12.92 ± 1.08 ^a^	11.06 ± 0.60 ^a^	5.64 ± 0.51 ^a^	0.08 ± 0.01 ^c^	93.12 ± 0.75 ^a^	0.54 ± 0.04 ^a^	2.37 ± 0.13 ^a^
PCE	10.15 ± 1.41 ^b^	9.21 ± 0.41 ^b^	4.17 ± 0.10 ^b^	0.19 ± 0.01 ^a^	92.45 ± 0.30 ^b^	0.47 ± 0.02 ^b^	2.18 ± 0.17 ^b^
LNE	11.20 ± 0.28 ^ab^	7.42 ± 0.53 ^c^	4.28 ± 0.13 ^b^	0.16 ± 0.01 ^b^	91.71 ± 0.10 ^c^	0.38 ± 0.04 ^c^	1.92 ± 0.03 ^c^

Note. PHE: Puerto Huallape Ecotype, PCE: Puerto Chinchipe Ecotype, LNE: Las Naranjas Ecotype. Different lowercase superscript letters indicate a significant difference according to Tukey’s test (*p* value = 0.00).

**Table 5:** Proximate composition of sour starch from different *Manihot esculenta* ecotypes.

Characteristic/Sample	Code		
	AQ25-0802/1	AQ25-0802/2	AQ25-0802/3
	PCE	PHE	LNE
Moisture (%)	8.47	10.71	7.67
Total protein (N × 6.25) (%)	0.48	0.48	0.66
Ether extract (%)	0.00	0.00	0.13
Crude fiber (%)	0.00	0.04	0.08
Ash (%)	0.26	0.10	0.28
Nitrogen-free extract (%)	90.79	88.67	91.18
Total dietary fiber (%)	2.30	1.40	1.10

**Table 6:** Thermal properties of sour starch from different cassava ecotypes (MEAN ± SD, n = 3).

Sour Starch	T_i_ (°C)	T_f_ (°C)	T_p_ (°C)	ΔH (J/g)
EPH	62.96 ± 1.74 ^a^	79.30 ± 1.14 ^a^	70.87 ± 0.61 ^a^	9.15 ± 1.81 ^a^
EPC	62.60 ± 3.40 ^a^	81.60 ± 1.64 ^a^	71.72 ± 0.44 ^a^	8.12 ± 0.77 ^b^
ELN	53.30 ± 2.91 ^b^	72.63 ± 2.52 ^b^	64.13 ± 0.37 ^b^	8.03 ± 1.72 ^c^

**Table 7:** Functional properties of sour starch from different cassava ecotypes (MEAN ± SD, n = 3).

Sour Starch	WSI (%)	WAI (g/g)
PHE	1.02 ± 0.04 ^a^	1.79 ± 0.03 ^c^
PCE	0.63 ± 0.03 ^b^	1.85 ± 0.04 ^b^
LNE	0.16 ± 0.01 ^c^	1.98 ± 0.04 ^a^

Note. PHE: Puerto Huallape Ecotype, PCE: Puerto Chinchipe Ecotype, LNE: Las Naranjas Ecotype. WSI: water solubility index, WAI: water absorption index. Different lowercase letters indicate a significant difference between means according to Tukey’s test (*p* value ≤ 0.05).

**Table 8:** Pasting parameters of sour starches from different cassava ecotypes.

Ecotypes	Peak Viscosity (cP)	Minimum Viscosity (cP)	Final Viscosity (cP)	Pasting Temperature (°C)	Breakdown (cP)	Setback (cP)
PHE	6502 ± 58. 81 ^a^	2543 ± 47.57 ^b^	3354 ± 173.675 ^b^	74.65 ± 0.48 ^a^	3961	819
PCE	6131 ±86.26 ^b^	2606.3 ± 56.36 ^b^	3257 ± 79.221 ^b^	74.833 ± 0.46 ^a^	3525	651
LNE	5671 ± 62.217 ^c^	4645 ± 14.45 ^a^	6248.667 ± 206.95 ^a^	69.617 ± 0.51 ^b^	1026	1604

Note. EPH: Puerto Huallape Ecotype; EPC: Puerto Chinchipe Ecotype; ELN: Las Naranjas Ecotype. Different lowercase letters within the same column indicate significant differences between ecotypes according to Tukey’s test (*p* ≤ 0.05).

## Data Availability

The authors confirm that the data supporting the findings of this study are available within the article.
